# The navel nanoethosomal formulation of gamma-oryzanol attenuates testicular ischemia/reperfusion damages

**DOI:** 10.1016/j.heliyon.2024.e28687

**Published:** 2024-04-06

**Authors:** Mobina Khormali, Mohammad Reza Farahpour

**Affiliations:** a Department of Basic Sciences, Faculty of Veterinary Medicine, Urmia Branch, Islamic Azad University, Urmia, Iran; b Department of Clinical Sciences, Faculty of Veterinary Medicine, Urmia Branch, Islamic Azad University, Urmia, Iran

**Keywords:** Testicular torsion, Reperfusion, Gamma-oryzanol, Nanoethosome, Antioxidant, Apoptosis

## Abstract

Testicular torsion reduces blood flow to testes and induces tissue ischemia. Antioxidant can have pivotal roles in alleviation of the effects of torsion/reperfusion. Gamma-oryzanol (γ-Oryzanol) has several pharmacological properties such as antioxidant and anti-apoptosis that can be used in this way. This study was conducted to evaluate the effects of nanoethosomal formulation of gamma-oryzanol (γ-Oryzanol-NEs) on testicular damages in a mouse model of ischemia/reperfusion damage. Following induction of ischemia/reperfusion, the mice were treated with γ-Oryzanol and γ-Oryzanol-NEs (6 mg/kg) in times of 3 h and 6 h. The expression of positive cells of TUNEL, superoxide dismutase (SOD), glutathione peroxidase (GPx), heat shock protein-70 (HSP70) and caspase 3 and histopathological parameters were assessed. The results showed higher expression of positive cells of TUNEL, HSP70 and caspase 3 and lower expressions of SOD and GPx in control mice compared with those treated with γ-Oryzanol-NEs (P = 0.001). The treatment with γ-Oryzanol-NEs could decrease pathological damages and the expression of positive cells of TUNEL, HSP70 and caspase 3 and increase the expressions of SOD and GPx. In conclusion, γ-Oryzanol-NEs could have the protective effects on torsion/reperfusion by decreasing apoptosis and increasing antioxidant status in a mouse model.

## 1 Introduction

Testicular torsion is an emergency status that may cause testicular ischemia and atrophy when the diagnosis is accompanied by delay [1]. Testicular torsion is known as a common urologic status in that testicular tissue, epididymis, and spermatic cord rotate around the longitudinal axis [2]. Testicular torsion reduces blood flow to testes and induces tissue ischemia. Reperfusion injuries are commonly observed following testes surgeries due to progressive damages in the activity and structure of the testes [3]. Following induction of ischemia, reactive oxygen species are accumulated as pathologic responses that result in infertility [4]. The species react with macromolecules and induce faults in cell function, DNA damage and apoptosis [5]. Production of reactive oxygen species causes damages in oxidative phosphorylation, increases mitochondrial dysfunction and induces a vicious cycle [6]. Cellular death due to the inflammation is one essential phases for starting the inflammation phase and caspase has a major role in it [7]. The emergency D‐torsion surgery is considered as a therapeutic option for testicular torsion, however, the reperfusion‐induced damages might be accompanied with endothelial cells injury, microcirculation disturbance and losing sever germ cells [8]. It has been reported beneficial effects of antioxidant and anti-inflammatory agents on ischemia/reperfusion damage-induced damages [9].

Gamma-oryzanol (γ-Oryzanol) has several pharmacological properties such as antioxidant, anti-inflammatory, anti-cancer, anti-diabetic, decreasing unpleasant menopausal symptoms, sugar and lipid [10]. It is known to have antioxidant activities [11,12] and ability of modulation in apoptosis [13,14]. Gamma-oryzanol has limitations in its physical structures for drug delivery that can be decreased with the help of nanotechnology science. The appropriate properties of nanocarriers for delivery are high capacity for drug loading, biocompatible, low systemic absorption and high dermal accumulation. Lipid vesicular systems are commonly utilized owing to their capability for carrying hydrophobic and hydrophilic drugs, safety, and biodegradability [15]. Ethosome is a new type of lipid vesicular carrier used for increasing delivery of different drugs [16,17].

γ-Oryzanol can be applied to attenuate testicular damages in ischemia/reperfusion damage due to its properties and ethosomes can be used for coating γ-Oryzanol. However, any study has not been evaluated the effects of nanoethosomal formulation of γ-Oryzanol on testicular damages in a mouse model of ischemia/reperfusion damage. This study aimed to evaluate nanoethosomal formulation of γ-Oryzanol (γ-Oryzanol-NEs) on testicular damages in a mouse model of ischemia/reperfusion damage by assessing superoxide dismutase (SOD), Glutathione peroxidase (GPx), heat shock protein-70 (HSP70 and caspase 3.

## 2 Materials and methods

### 2.1 Materials

The Soy lecithin was purchased from Lipoid GmbH, Ludwigshafen, Germany. Gamma-oryzanol was a gift from Tsuno Rice Fine Chemicals Co., Ltd, Japan. Miglyol® 812 were obtained from Gattefosse (St-Priest, France). Other agents were prepared from Sigma Aldrich Company (Chemie, Steinheim, Germany).

### 2.2 Preparation of nanoethosomes

Nanoethosomes (NEs) were synthesized with the help of a modified ethanol injection method by soy lecithin as a surfactant as reported by others [18]. In summary, the temperature was controlled and fixed in 65 ^°^C bath with the help of “Heidolph Hei-Tec” magnetic stirrer with Pt 1000 temperature sensor, 350 mg of soy lecithin, 30 mg of γ-Oryzanol, was dissolved entirely in 10 mL of ethanol as lipid/γ-Oryzanol solution, and slowly administrated with 1 mL/min flow rate into the 30 mL of heated distilled water in temperature of 65 ^°^C by mixing with a homogenizer (Silent Crusher M, Heidolph, Germany) (20,000 rpm). The homogenization step was followed for 15 min after ending the lipid/γ-Oryzanol solution to achieve γ-Oryzanol-NEs in sub 100 nm size. The empty nanoethosomal formulation (NEs without γ-Oryzanol), and Single nanoethosomal formulations (γ-Oryzanol -NEs) were also prepared with the same method.

### 2.3 Preparation of oil-mediated free formulation

To prepare an oil-mediated free formulation, 6 mg of γ-Oryzanol were dissolved into 6 mL of Miglyol 812 and used for further considerations.

### 2.4 Characterization of prepared ethosomes

Dilutions of ethosomes were performed 1:100 to prevent the accumulation of nanoparticles. Particle size and zeta potential of nanoethosomal formulations were assessed by laser diffraction technique (Nano ZS, Malvern, UK). Scanning electron microscope (SEM) (MIRA3 TESCAN, UK, LEO 84 1430VP, UK & Germany) was conducted at 15 kV [19].

### 2.5 Encapsulation efficacy

The amount of loaded γ-Oryzanol was estimated to evaluate the encapsulation efficacy (EE%) and drug loading capacity (DLC%) indexes [20,21]. To separate the unload drug, an ultra-filtration technique was performed using Amicon® Ultra-Centrifugal Filter with 30 KD MWCO (Merck Millipore, Germany). Briefly, 2 mL of sample was diluted with ethanol and the final sample had 40% ethanol for confidence of the complete solubilization of the unloaded γ-Oryzanol. The diluted sample was then placed into the upper chamber of the filter and centrifuged with the speed of 4000 rpm for 10 min (Rotofix 32 A, Hettich, Germany) and the accumulated solution into the down chamber was collected and stored in the refrigerator till analysis time. To evaluate the unload γ-Oryzanol ultraviolet–visible spectroscopy (UV–Vis) method using Ultrospec 2000 Pharmacia Biotech, (Cambridge, England) UV–V instrument was performed at 319 and 285 nm, respectively.

### 2.6 Toxicity

Acute toxicity was conducted to elucidate safety of γ-Oryzanol as outlined by others [22]. Thirty-five mice were grouped into eight groups (n = 5) and received different doses of γ-Oryzanol (5, 10 and 20 mg/kg) and its ethosome (5, 10 and 20 mg/kg) while control groups received vehicles for 14 days. Mortality, food, water intake, diarrhea and other toxicity signs were monitored until two weeks after the last administration. Any diarrhea was not observed up to 10 mg/kg for the free and encapsulated forms, while diarrhea and lethargy were observed in 20 mg/kg. Thus, concentration of 6 mg/kg was used in the current study.

### 2.7 Experimental groups, drug administration and animal care

In this study, 54 male BALB/c mice of 4 weeks of age weighting 20 ± 2 g were prepared. The mice were kept in cages on sawdust bedding at a constant temperature of 24 ± 2 ^°^C under 12-h periods of light-dark exposure. The mice had free access to standard food and water. The Ethical Committee of Islamic Azad University approved all the protocols used for the care and treatment of mice (IAUU2023/14/March 2022). The mice were adapted to the environment for one week and subjected to 3 h and 6 h unilateral testicular torsion. The mice were grouped into six groups control in times of 3 h (n = 9) and 6 h (n = 9) and treated with γ-Oryzanol in times of 3 h (n = 9) and 6 h (n = 9) and γ-Oryzanol-NEs in times of 3 h (n = 9) and 6 h (n = 9). Following induction of testis torsion, the mice were treated with 6 mg/kg of γ-Oryzanol and γ-Oryzanol-NEs in times of 3 h and 6 h only once. Control mice were treated with saline.

### 2.8 Surgical procedure of torsion and reperfusion induction

Torsion and reperfusion was conducted as reported by others [2]. In summary, after induction of anesthesia with the help of ketamine and xylazine, the scrotum was entered by a left inguinoscrotal incision. Firstly, tunica vaginalis was opened and torsion was created by rotating the left testis 720° clockwise, and then the torsion was kept with the help of suturing the testicle for the scrotal wall with silk 3–0. In end, the incision was closed and allowed rats to recovery. Following the induction of torsion, reperfusion was conducted. Re-anesthesia was conducted and reperfusion was conducted with the help of counter-rotation and restoring of the testicular gubernacular stump for the scrotal gubernacular stump. The mice were evaluated for recirculation of blood flow. The testes were placed into the scrotum.

### 2.9 Histopathological study and spermatogenesis evaluation

In the end of the surgical procedure, the samples of testicular tissues were immediately ﬁxed in 10% formaldehyde phosphate buffer for 48 h. The testicular tissue samples were dehydrated, cleared in xylene and embedded in parafﬁn. Sections (5 μm) were prepared, deparafﬁnized and stained using hematoxylin and eosin staining technique and assessed under blindfold conditions with standard light microscopy (Olympus, Germany). Mean seminiferous tubule diameter (MSTD) was reported as μm. To assess the histologic changes in germinal cells and seminiferous tubules, the Cosentino's scoring system was applied [23].

### 2.10 Immunofluorescent staining

Immunofluorescence staining was performed based on reports of other studies [24]. In summary, tissue samples were prepared, Paraffin-embedded tissue sections were dewaxed, rehydrated and immersed in citrate buffer (pH 7.4) for 20 min for antigen retrieval. Slides were washed with TBS plus 0.03% Triton X-100, blocked in 10% normal serum or with 1% BSA in TBS for 2 h at room temperature. The samples were investigated for TUNEL, SOD, GPx, HSP-70 and caspase. The used antibodies included TUNEL (E-CK-A334, Elabscience Company), SOD (ab51254, Abcam Company), GPx (sc-58361, Santa Cruz Company), HSP-70 (sc-66048, Santa Cruz Company), and caspase 3 (D3R6Y) Rabbit mAb #14220, Cell Signaling), Goat Anti-Mouse IgG (E-AB-1011, Elabscience Company) and Goat Anti-Rabbit IgG (E-AB-1014, Elabscience Company).

### 2.11 Data analysis

The data were analyzed for normality with the help of Kolmogorov–Smirnov test in SPSS software (version 23) and analyzed with the help of one-way ANOVA. Post-hoc comparisons were conducted with the help of Duncan and P < 0.05 was considered significant.

## 3 Results

**Fig. 1:**
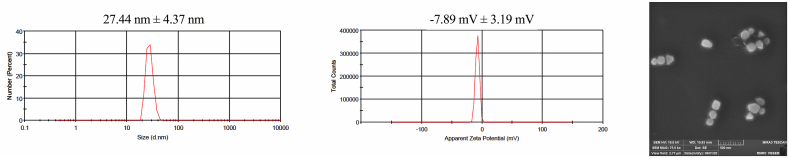
Size (left), zeta potential (center), and scanning electron microscopy (right) of gamma-oryzanol loaded nanoethosomes.

As shown in Fig. 1, the average particle size distribution of γ-Oryzanol is 27.44 nm. On other hand, the surface charge of the NEs were as follows: the γ-Oryzanol represent −7.89 mV for zeta potential. SEM analysis showed spherical morphology for prepared NEs and confirmed sizes (Fig. 1). The EE% of γ-Oryzanol-NEs was 96.11 ± 4.14%. On other hand, DLC% of the γ-Oryzanol into the NEs was 13.84 ± 1.47. The negatively close zero surface charge of particles is also promising for enhancing transdermal delivery [25,26]. Zero surface charge is necessary for colloidal stability. The formulations were introduced in a gel-medium immediately after preparation to minimize dynamic changes in the particles and stabilize the NEs. DLC% of liposomes are usually not more than 10% [27]. In this case, more than 13% of DLC% was reported due to higher hydrophobicity of γ-Oryzanol, addition hydrogen/oxygen bonds of γ-Oryzanol structure, which could form hydrogen bonds with the polar head group of lecithin [25,27] in the outer layer of the ethosomal system. These consequences could be the reason for the low negative zeta potential value and the higher ethosomal system of DLC%.

### 3.1 Histopathologic results

**Fig. 2:**
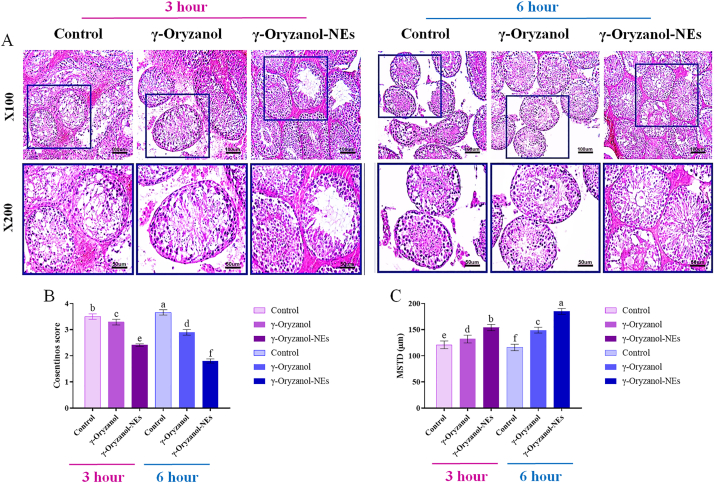
(A) Light microscopy of testicular tissue in experimental groups (hematoxylin and eosin staining - 100 × ). (B) Histological evaluation of the testes using Cosentino's scores (B) and MSTD values (C) in times of 3 and 6 h after. Superscripts (a–e) show significant differences between groups in the same time.

The protective effects of γ-Oryzanol on testis tissue are shown in Fig. 2. The results were analyzed using H&E staining. Normal histological architecture of seminiferous tubules with normal arrangement of germinal epithelium and no evidence of hemorrhage, inflammation or any other abnormalities were observed in sham-operated group samples (Fig. 2a). In control group, severe disruption and disorganization of seminiferous tubules and desquamation in germinal epithelium were seen (Fig. 2b).

Fig. 2 shows histological evaluation of the testes using Cosentino's scores (a) and MSTD values (b) in times of 3 and 6 h. The average of Cosentino score in the treatment groups was decreased compared to the control group at 3 h and in the nano-oryzanol group it was significant. The decreased cosentino score in the nano-oryzanol treatment group is significant compared to the oryzanol treatment group at 3 h. The mean of Cosentino score in the treatment groups (oryzanol and nano-oryzanol) was decreased compared to the control group at 6 h, which is significant in both treatment groups at 6 h (oryzanol and nano-oryzanol). The difference in cosentino score in the nano-oryzanol treatment group compared to the oryzanol treatment group at 6 h is also significant.

The mean of diameter of the seminiferous tubules in the treatment groups was increased compared to the control group at 3 h, and this increase is seen only in the nano-oryzanol group. The average diameter of the seminiferous tubules in the treatment groups (oryzanol and nano-oryzanol) at 6 h has increased compared than the control group at 6 h, and this increase is significant in both treatment groups. The average diameter of the seminiferous tubules in the nano-oryzanol treatment group was increased significantly compared with the oryzanol treatment group at 6 h.

### 3.2 TUNEL results

**Fig. 3:**
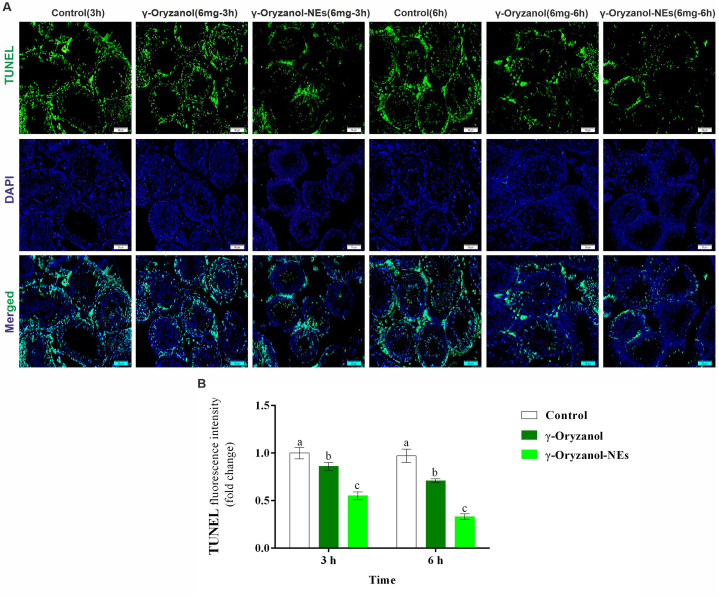
(A) The effects of γ-Oryzanol and γ-Oryzanol-NEs on the expression of TUNEL in immunofluorescent staining and (B) software analyses for pixel-based intensity of chromogen (positive reaction for TUNEL). See the lower expressions of TUNEL in γ-Oryzanol and γ-Oryzanol-NEs treated groups versus the control group. The results are expressed as the mean ± SD. Non-similar letters on figures (a–d) show significant differences (*P* < *0.05*) between groups.

Fig. 3 illustrates the results for the effects of γ-Oryzanol and γ-Oryzanol-NEs on the expression of TUNEL in immunofluorescent staining. The results showed that the number of positive apoptotic cells in control mice was significantly higher in times of 3 h and 6 h compared with those treated with γ-Oryzanol and γ-Oryzanol-NEs (P = 0.001). The results showed the expression of positive cells of apoptotic was significantly higher 14.00% and 35.00% compared with the mice treated with γ-Oryzanol and γ-Oryzanol-NEs in time of 3 h (P = 0.001) and 18.00% and 60.00% in time of 6 h, respectively. The positive cells were significantly higher in the mice treated with γ-Oryzanol compared with those treated with γ-Oryzanol-NEs in both times.

### 3.3 The expressions of SOD and GPx

**Fig. 4:**
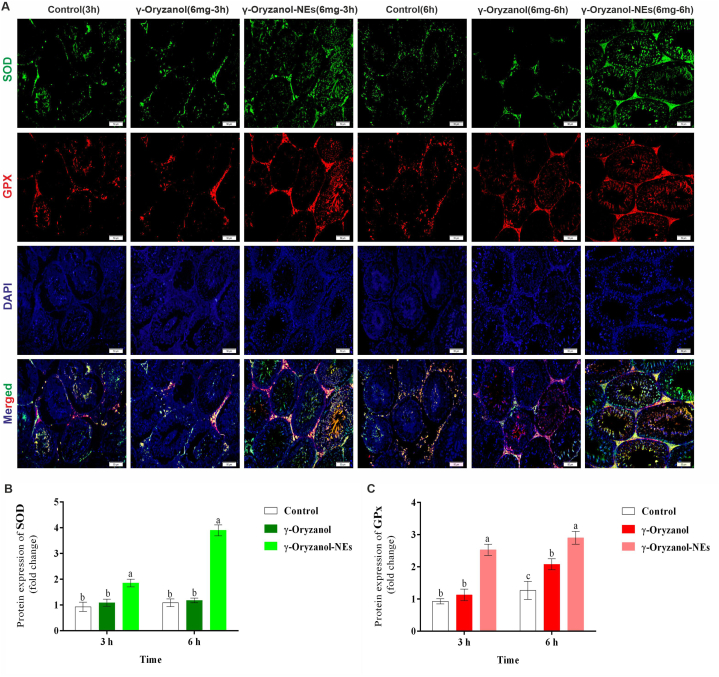
(A) The effects of γ-Oryzanol and γ-Oryzanol-NEs on the expression of SOD and GPx in immunofluorescent staining and (B) software analyses for pixel-based intensity of chromogen (positive reaction for SOD and GPx). See the higher expressions of SOD and GPx in γ-Oryzanol and γ-Oryzanol-NEs treated groups versus the control group. The results are expressed as the mean ± SD. Non-similar letters on figures (a–d) show significant differences (*P* < *0.05*) between groups.

The expressions of SOD and GPx in different groups are shown in Fig. 4. The results did not show significant differences between γ-Oryzanol and control mice on times of 3 h (P = 0.562) and 6 h (P = 0.854). The expression of positive cells of SOD was significantly higher in γ-Oryzanol-NEs mice by 50.00% and 3 times in times of 3 h and 6 h, respectively (P = 0.001).

The results for the expression of GPx did not show significant differences between the control and γ-Oryzanol at a time of 3 h (P = 0.814). However, the expression of positive cells of GPx was significantly higher 63.00% in γ-Oryzanol-NEs compared with control mice in a time of 3 h. In addition, the expression of positive cells of GPx was significantly higher 39.00% and 56.00% in γ-Oryzanol and γ-Oryzanol-NEs mice compared with control mice in a time of 6 h, respectively. The results showed significant differences between γ-Oryzanol and γ-Oryzanol-NEs for GPx and SOD in all times.

### 3.4 The expressions of caspase 3 and HSP-70

**Fig. 5:**
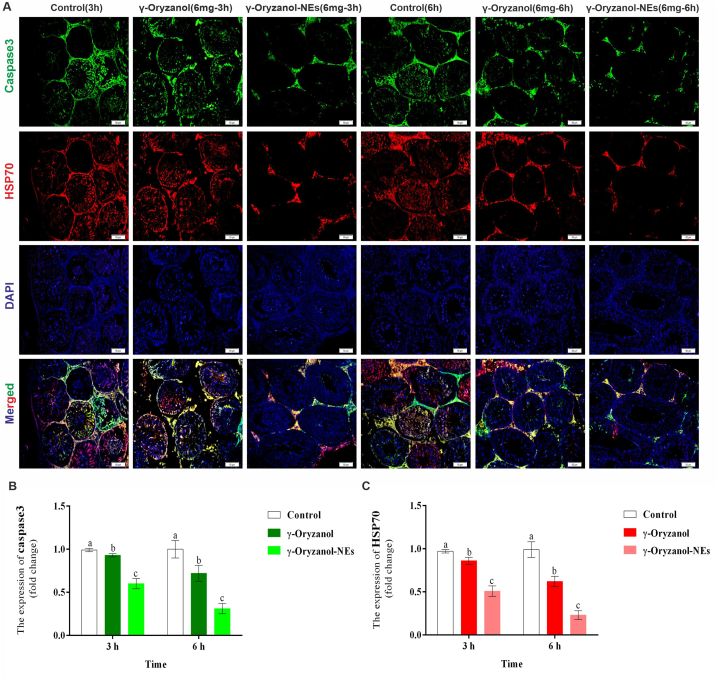
(A) The effects of γ-Oryzanol and γ-Oryzanol-NEs on the expression of caspase 3 and HSP-70 in immunofluorescent staining and (B) software analyses for pixel-based intensity of chromogen (positive reaction caspase 3 and HSP-70). See the lower expressions of caspase 3 and HSP-70 in γ-Oryzanol and γ-Oryzanol-NEs treated groups versus the control group. The results are expressed as the mean ± SD. Non-similar letters on figures (a–d) show significant differences (*P* < *0.05*) between groups.

Fig. 5 depicts the effects of γ-Oryzanol and γ-Oryzanol-NEs on the expression of caspase 3 and HSP-70 in immunofluorescent staining. The results showed lower expression of positive cells of HSP70 in γ-Oryzanol and γ-Oryzanol-NEs compared with control mice in both times (*P* < *0.05*). The results showed significant differences between γ-Oryzanol and γ-Oryzanol-NEs in both times. The results showed that expression of caspase 3 was significantly lower in the mice treated with γ-Oryzanol and γ-Oryzanol-NEs compared with control mice in both times (P = 0.001).

## 4 Discussion

This study was conducted to evaluate the effects of γ-Oryzanol and γ-Oryzanol-NEs on testicular damages in a mouse model of ischemia/reperfusion damage. Testicular torsion is known as a pathologic and emergency status requiring surgery and it can cause damages in testes in longer ischemic condition [28]. The results showed that positive cells in TUNEL staining was higher in control mice. TUNEL is used as a test for the identification of apoptotic DNA fragmentation, and quantify apoptotic cells and also detection of excessive DNA breakage in individual cells. It has been accepted that apoptotic cell death has a pivotal role in limiting testicular germ cell population after testicular ischemia/reperfusion damage and its dysregulation can be associated with infertility. Germ cell homeostasis following testicular ischemia/reperfusion relies on a balance between cell production from a constantly renewing population of testicular stem cells, germ cell differentiation and programmed cell death [29]. The increase in apoptosis of the primary spermatocytes is a mechanism to lose germ cells in the ischemic testis [30]. It was reported the increase in apoptosis in the contralateral testis after ischemia [31]. Tunnel-positive cells were higher in control mice confirm induction of apoptosis in cells. However, γ-Oryzanol and γ-Oryzanol-NEs could decrease apoptosis in the mice. The results agree with other studies for the effects of γ-Oryzanol and γ-Oryzanol-NE in suppressing apoptosis [13,14]. It could be attributed to the effects of γ-Oryzanol in prevention of oxidation [13,14]. In the current study, γ-Oryzanol and γ-Oryzanol-NEs increased antioxidant activity and decreased the expression of caspase as a molecule involved in apoptosis. This result shows that γ-Oryzanol and γ-Oryzanol-NEs can decrease apoptosis and higher effects of γ-Oryzanol-NEs compared with γ-Oryzanol could be attributed to the protective effects of NEs.

In the current study, the expression of SOD-GPx-positive cells were significantly lower in the control mice. Indeed, testicular torsion reopening of the blood flow following massive ischemia increases molecular oxygen and over-generation of reactive oxygen species in ischemic tissue [28]. On the other hand, high levels of reactive oxygen species cannot be cleared with the help of endogenous antioxidants due to imbalance in oxidant and antioxidant status [32]. During testicular torsion, an imbalance between oxygen demand and supply is increasing which results in tissue hypoxia. This hypoxia causes lipid peroxidation, inflammation and apoptosis in the testis that finally cause cellular death [33]. The results did not show strong effects of γ-Oryzanol on the increase in the expression of SOD and GPx, however, γ-Oryzanol-NEs had higher effects. The results are in agreement with previous studies for antioxidant activity of γ-Oryzanol [11,34]. Seemingly, loading γ-Oryzanol into NEs protects them for antioxidant activities and helps them to exhibit maximum efficiency.

The results also showed that expression of caspase-3 was significantly higher in control mice. Caspase-3 has a major role in promoting apoptosis and the increase in the expression of caspase-3 is considered as an marker for apoptosis. The increase in the expression of caspase-3 is commonly observed following testicular torsion and reperfusion [35]. As mentioned, apoptosis occurs during ischemia/reperfusion. In this study, the results for TUNEL and caspase are parallel. It exhibits that γ-Oryzanol and γ-Oryzanol-NEs inhibit apoptosis via decreasing the expression of caspase-3.

The results also showed higher positive-cell of HSP70 in control mice that are in agreement with other studies for higher expression of HSP-70 after testicular torsion [36]. However, the treatment with γ-Oryzanol and γ-Oryzanol-NEs could decrease the expression of HSP-70. The mechanism of γ-Oryzanol and γ-Oryzanol-NEs in decreasing the expression of positive-cell of HSP-70 is not clear, however, γ-Oryzanol-NEs had higher effects which could be attributed to its coating with NE.

Histopathological results confirmed that γ-Oryzanol decreases the testis damages, which could be attributed to their effects on oxidative stress. Indeed, oxidant-antioxidant system of the testicular tissue is changed during torsion that induces changes in activities of key enzymes of hexose monophosphate, glucose 6 phosphate dehydrogenase parallel with changes in glutathione content [37]. The mechanism of γ-Oryzanol on oxidative metabolism and enzymes needs further investigations for its effects on enzymes.

## 5 Conclusions

In conclusion, γ-Oryzanol-NEs can attenuate adverse effects of torsion/reperfusion on testes by decreasing apoptosis and improving antioxidant status. The results are promising and can be used for the treatment and prevention of damage in torsion/reperfusion. This study was conducted on a rodent model that is a major limitation for it but the results are novel and promising. The results can be used for human clinical following future clinical and conducting mechanistic studies.

## Funding

We would like to thank glory god, the Lord of Mercy, almighty for making this work possible. This research did not receive any specific grant from public, commercial, or not-for-profit funding agencies.

## CRediT authorship contribution statement

**Mobina Khormali:** Validation, Project administration, Funding acquisition, Conceptualization. **Mohammad Reza Farahpour:** Writing – review & editing, Writing – original draft, Visualization, Validation, Supervision, Software, Resources, Project administration, Methodology, Investigation, Funding acquisition, Formal analysis, Data curation, Conceptualization.

## Declaration of competing interest

The authors declare that they have no conflict of interest.

## References

[1] Tanriverdi H.I., Şenel U., Gevrek F., Akbaş A. (2021). Protective effect of famotidine on ischemia–reperfusion injury following testicular torsion in rats. J. Pediatr. Urol..

[2] Shamsi-Gamchi N., Razi M., Behfar M. (2018). Testicular torsion and reperfusion: evidences for biochemical and molecular alterations. Cell Stress Chaperones.

[3] Kazemi-Darabadi S., Asadpour R., Shahbazfar A.A., Alizadeh S. (2019). Veterinary Research Forum.

[4] Doğan G., İpek H. (2020). The protective effect of Ganoderma lucidum on testicular torsion/detorsion-induced ischemia-reperfusion (I/R) injury. Acta Cir. Bras..

[5] Juan C.A., Pérez de la Lastra J.M., Plou F.J., Pérez-Lebeña E. (2021). The chemistry of reactive oxygen species (ROS) revisited: outlining their role in biological macromolecules (DNA, lipids and proteins) and induced pathologies. Int. J. Mol. Sci..

[6] Niu X., Pu S., Ling C., Xu J., Wang J., Sun S. (2020). lncRNA Oip5‐as1 attenuates myocardial ischaemia/reperfusion injury by sponging miR‐29a to activate the SIRT1/AMPK/PGC1α pathway. Cell Prolif..

[7] Hirao H., Nakamura K., Kupiec-Weglinski J.W. (2022). Liver ischaemia–reperfusion injury: a new understanding of the role of innate immunity. Nat. Rev. Gastroenterol. Hepatol..

[8] Talebi H., Farahpour M.R. (2019). Testicular torsion and reperfusion: germ cell DNA damage and development. Andrologia.

[9] Afolabi O., Anyogu D., Hamed M., Odetayo A., Adeyemi D., Akhigbe R. (2022). Glutamine prevents upregulation of NF-kB signaling and caspase 3 activation in ischaemia/reperfusion-induced testicular damage: an animal model. Biomed. Pharmacother..

[10] Ramazani E., Akaberi M., Emami S.A., Tayarani-Najaran Z. (2021). Biological and pharmacological effects of gamma-oryzanol: an updated review of the molecular mechanisms. Curr. Pharmaceut. Des..

[11] Minatel I.O., Francisqueti F.V., Corrêa C.R., Lima G.P.P. (2016). Antioxidant activity of γ-oryzanol: a complex network of interactions. Int. J. Mol. Sci..

[12] Hajilou H., Farahpour M.R., Hamishehkar H., Polycaprolactone nanoﬁber coated with chitosan and Gamma oryzanol functionalized as a novel wound dressing for healing infected wounds Int. J. Biol. Macromol.16420202358–2369. 10.1016/j.ijbiomac.2020.08.079 32791277

[13] Huang L., Jiang W., Zhu L., Ma C., Ou Z., Luo C. (2020). γ-Oryzanol suppresses cell apoptosis by inhibiting reactive oxygen species-mediated mitochondrial signaling pathway in H2O2-stimulated L02 cells. Biomed. Pharmacother..

[14] Zhu L., Xiang S., Ou Z., Ma C., Yi X., Huang L. (2019). Gamma-oryzanol prevents ethanol-induced liver injury by ameliorating oxidative stress and modulating apoptosis-related protein expression in mice. J. Funct.Foods.

[15] Wang G., Wang J., Wu W., Tony To S.S., Zhao H., Wang J. (2015). Advances in lipid-based drug delivery: enhancing efficiency for hydrophobic drugs. Expet Opin. Drug Deliv..

[16] Touitou E., Dayan N., Bergelson L., Godin B., Eliaz M. (2000). Ethosomes—novel vesicular carriers for enhanced delivery: characterization and skin penetration properties. J. Contr. Release.

[17] Ghanbarzadeh S., Arami S. (2013). Enhanced transdermal delivery of diclofenac sodium via conventional liposomes, ethosomes, and transfersomes. BioMed Res. Int..

[18] Zeinali M., Abbaspour-Ravasjani S., Soltanfam T., Paiva-Santos A.C., Babaei H., Veiga F. (2021). Prevention of UV-induced skin cancer in mice by gamma oryzanol-loaded nanoethosomes. Life Sci..

[19] Asadollahi L., Mahoutforoush A., Dorreyatim S.S., Soltanfam T., Paiva-Santos A.C., Peixoto D. (2022). Co-Delivery of erlotinib and resveratrol via nanostructured lipid Carriers: a synergistically promising approach for cell proliferation prevention and ROS-Mediated apoptosis activation. Int. J. Pharm..

[20] Mahoutforoush A., Abbaspour-Ravasjani S., Nazarpak M.H., Hamishehkar H., Solouk A. (2021). Novel decorated nanostructured lipid carrier for simultaneous active targeting of three anti-cancer agents. Life Sci..

[21] Mahoutforoush A., Solouk A., Hamishehkar H., Nazarpak M.H., Abbaspour-Ravasjani S. (2021). Novel decorated nanostructured lipid carrier for simultaneous active targeting of three anti-cancer agents. Life Sci..

[22] Guideline P-BT (2001). OECD guideline for the testing of chemicals. The Hershberger.

[23] Cosentino M.J., Nishida M., Rabinowitz R., Cockett A.T. (1985). Histological changes occurring in the contralateral testes of prepubertal rats subjected to various durations of unilateral spermatic cord torsion. J. Urol..

[24] Liao F., Chen L., Luo P., Jiang Z., Chen Z., Wang Z. (2020). PC4 serves as a negative regulator of skin wound healing in mice. Burns Trauma.

[25] Gillet A., Compère P., Lecomte F., Hubert P., Ducat E., Evrard B. (2011). Liposome surface charge influence on skin penetration behaviour. Int. J. Pharm..

[26] Adib Z.M., Ghanbarzadeh S., Kouhsoltani M., Khosroshahi A.Y., Hamishehkar H. (2016). The effect of particle size on the deposition of solid lipid nanoparticles in different skin layers: a histological study. Adv. Pharmaceut. Bull..

[27] Zeinali M., Abbaspour-Ravasjani S., Soltanfam T., Paiva-Santos A.C., Babaei H., Veiga F. (2021). Prevention of UV-induced skin cancer in mice by gamma oryzanol-loaded nanoethosomes. Life Sci..

[28] Jafari A., Ghasemnejad-Berenji H., Nemati M., Ghasemnejad-Berenji M. (2021). Topiramate: a novel protective agent against ischemia reperfusion-induced oxidative injury after testicular torsion/detorsion. Am. J. Emerg. Med..

[29] Kanter M. (2010). Protective effects of melatonin on testicular torsion/detorsion-induced ischemia–reperfusion injury in rats. Exp. Mol. Pathol..

[30] Turner T., Tung K.S., Tomomasa H., Wilson L.W. (1997). Acute testicular ischemia results in germ cell-specific apoptosis in the rat. Biol. Reprod..

[31] Sukhotnik I., Miselevich I., Lurie M., Nativ O., Coran A.G., Mogilner J.G. (2005). The time relationship between ipsilateral testicular ischemia and germ cell apoptosis in the contralateral testis in rat. Pediatr. Surg. Int..

[32] Bozlu M., Acar D., Cayan S., Aktas S., Tunckiran A. (2009). Protective effect of trapidil on long-term histologic damage in a rat model of testicular ischemia-reperfusion injury. World J. Urol..

[33] Shimizu S., Tsounapi P., Dimitriadis F., Higashi Y., Shimizu T., Saito M. (2016). Testicular torsion–detorsion and potential therapeutic treatments: a possible role for ischemic postconditioning. Int. J. Urol..

[34] Toorani M.R., Golmakani M.-T. (2022). Effect of triacylglycerol structure on the antioxidant activity of γ-oryzanol. Food Chem..

[35] Gazia M.A. (2020). Histological study on the possible ameliorating effect of platelet rich plasma on ischemia/reperfusion injury in testicular torsion model in adult albino rat. Egypt. J. Histol..

[36] Sönmez M., Ozdemir Ş., Guzel M., Kaymak E. (2017). The ameliorative effects of vinpocetine on apoptosis and HSP-70 expression in testicular torsion in rats. Biotech. Histochem..

[37] Elshaari F., Elfagih R., Sheriff D., Barassi I. (2011). Oxidative and antioxidative defense system in testicular torsion/detorsion. Indian J. Urol: IJU: J. Urol. Soc. India.

